# Measurement of Absorption and Scattering With an Integrating Sphere Detector: Application to Microalgae

**DOI:** 10.6028/jres.114.006

**Published:** 2009-04-01

**Authors:** A. K. Gaigalas, Hua-Jun He, Lili Wang

**Affiliations:** Biochemical Science Division, National Institute of Standards and Technology, Gaithersburg, MD 20899

**Keywords:** absorbance, integrating sphere detector, microalgae, scattering

## Abstract

A spectrometer with an integrating sphere (IS) detector was used to measure the absorbance due to scattering and absorption. Analysis of the measurement process showed that two measurements of the absorbance, one with the cuvette placed in the normal spectrometer position, and the second with the cuvette placed next to the entrance aperture of the IS detector, provide enough information to separate the contributions from scattering and molecular absorption. Measurements were carried out with mixtures of microsphere and chromophore solutions. Two cases were examined: microspheres suspended in an aqueous fluorescein solution, and microspheres suspended in an aqueous holmium oxide solution. In both cases, the proposed measurement model gave results which were in good agreement with the expected response. Measurements on microalgae suspensions yielded a molecular absorption contribution and a scattering contribution. The scattering contribution had significant spectral structure which was inversely related to the molecular absorption contribution. The absorption and scattering contributions may provide independent information on the status of chlorophyll molecules and the structure of chloroplasts in microalgae.

## 1. Introduction

The measurement of absorption and scattering in a turbid environment is an important problem. Such measurements are used to characterize impurities in water [1] and to characterize aquatic particles [2]. In these applications, a spectrometer with an integrating sphere detector (IS) is ideal since the IS can reduce the effect of scattering and enhance the effect due to molecular absorption. Description of the use of IS with a cuvette holder inside the IS was given by Nelson [3]. A description of the IS with an internal sample is given by Labsphere [4]. An approach that places samples outside the IS was also described [5]. Although the IS with an internal sample seems to provide the least sensitivity to scattering, this arrangement results in an non-ideal IS [6] and has problems with stirring and temperature control. An IS with the sample filling the complete volume of the IS have been described in the literature [7,8]. This later technique is not practical for biological samples with requirements for small sample volume. Microalgae cultivation is an important area where absorption measurements will gain in importance. The absorption spectra of chlorophyll in plants is a rich source of information about the state of the microorganism [9]. In this work, we develop a more general and systematic model of the IS measurement process [10] and the interpretation of the resulting data. This work applies the measurement model to the measurement of absorbance in mixtures of microspheres and chromophores and to a suspension of microalgae.

## 2. Interpretation of Integrating Sphere (IS) Measurements

**Fig. 1:**
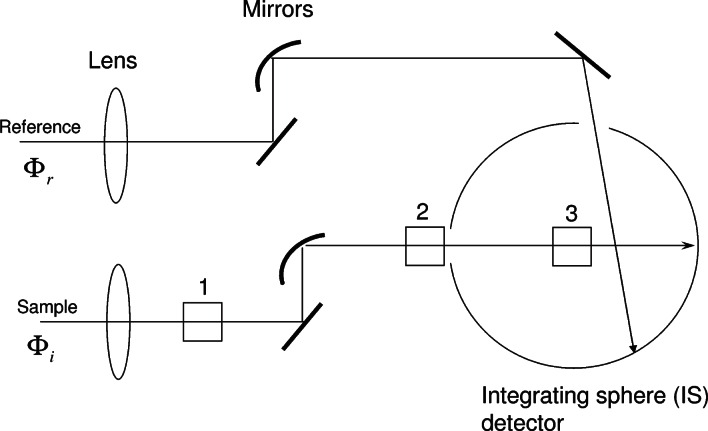
A schematic of the Perkins Elmer dual beam Lambda 850 spectrophotometer sample holders. Holder 1 represents the normal cuvette holder. Holder 2 places the cuvette in front of the entrance port of the integrating sphere (IS) detector. Holder 3 places the cuvette inside the IS and is not used in this study. For all cuvette holders, the same reference beam enters the IS detector through a reference port and hits the wall of the IS detector. In practice, the spectrometer is auto zeroed with all holders empty.

The proposed method for measuring the scattering and molecular absorption utilizes a spectrophotometer with an integrating sphere (IS) detector. For clarity, the measurement will be described with a specific instrument—the Perkin Elmer Lambda 850^1^. Figure 1 shows the paths of the sample and reference beams used in the measurement of absorbance. The two paths are very similar; both end on the wall of the IS detector. The path of the sample beam contains three mechanical holders for a cuvette labeled 1, 2, and 3 in Fig. 1. Two cuvette holders, 1 and 2, are located outside of the IS detector while the cuvette holder 3 is located inside the IS detector. The lens and the mirrors shape the sample beam so that it passes unobstructed through the cuvette holders. Therefore, two measurements can be performed with the cuvette placed outside the IS, and the third measurement with the cuvette inside the IS. In the following, we discuss a model of the response of the IS detector for the case where the sample is placed in cuvette holders 1 and 2. The case where the sample is placed in cuvette holder 3 is not treated. The response in holder 3 is complicated by the increased sensitivity to fluorescence and the inability to stir the suspension in holder 3. The use of holder 3 for measurement of fluorescence quantum yield has been described previously [11].

### 2.1 Response of the IS Detector With Samples in Cuvette Holder 1

An incident flux, Φ*_i_* (W-Watts), passes through the cuvette placed in holder 1, enters the IS through the sample aperture of area *A_i_* (m^2^), and hits the wall of the IS. A reference flux, Φ*_r_* (W), enters the IS through a reference aperture of area *A_r_* (m^2^) and hits the IS wall without passing through the cuvette. The reference beam will enter the IS, hit the IS wall and undergo the first reflection. A baffle inside the IS prevents the detector from seeing the first reflection of the incident reference beam. The reference light beam undergoes many more reflections resulting in an average flux, $\Phi_{r}^{s}$, incident on the IS surface. The average flux will be written as

$$\Phi_{r}^{s}=M_{ref}\Phi_{r}.$$ (1)

The symbol *M_ref_* represents the magnification of the incident reference flux due to the many reflections on the IS wall. Neglecting the presence of the baffle, the ideal IS behavior leads to the following expression for the magnification *M_ref_* = *ρ* (1 − *f*) / (1 − *ρ* (1 − *f*)). The constant *f* is defined as the ratio of areas given by *f* = (*A_i_* + *A_r_* + *A_d_*)/*A_s_* where *A_d_* and *A_s_* are the areas of the detector aperture and total sphere surface respectively. The average reflectance of the material lining the surface of the IS is given by *ρ* [12]. The photomultiplier (PM) detector is mounted flush with the surface of the IS. Therefore the PM detector response will be proportional to the average flux incident per unit surface area of the IS multiplied by the detector area. Explicitly, the detector signal, *D_r_*, will be given by Eq. (2)

$$D_{r}=R\left(\lambda \right)\Phi_{r}M_{ref}\frac{A_{d}}{A_{s}}\equiv E\left(\lambda \right)M_{ref}\Phi_{r}$$ (2)

where *R*(*λ*) is the radiant sensitivity of the photomultiplier cathode. The ratio of the detector area to the area of the entire IS is a constant which can be combined with the radiant sensitivity to give overall detection efficiency *E*(*λ*). It will be assumed that the dependence of the detection efficiency on wavelength comes mainly from the radiant sensitivity of the photomultiplier cathode. According to the manufacturer, the reflectance of the IS surface varies by less than 1 % over the spectral region considered in this work. This variation has a weak dependence on wavelength and may introduce a systematic uncertainty in the final result.

An equation similar to Eq. (2) can be written relating the detector response, *D_i_* to the incident flux, Φ*_i_* entering through the sample port

$$D_{i}=R\left(\lambda \right)\Phi_{i}M_{inc}\frac{A_{d}}{A_{s}}\equiv E\left(\lambda \right)M_{inc}\Phi_{i}.$$ (3)

The subscript on the magnification factor indicates that the magnification may be slightly different for the flux entering through the sample port to that for the flux entering through the reference port. The “autozero” function of the spectrometer is used to compensate for any differences in the IS detector response between the reference and sample beams in the case where there is no sample in the cuvette holder. We will assume that the detection efficiency is identical for both cases, so that the “autozero” function insures the equality given by Eq. (4) below

$$M_{inc}\Phi_{i}=M_{ref}\Phi_{r}.$$ (4)

**Fig. 2:**
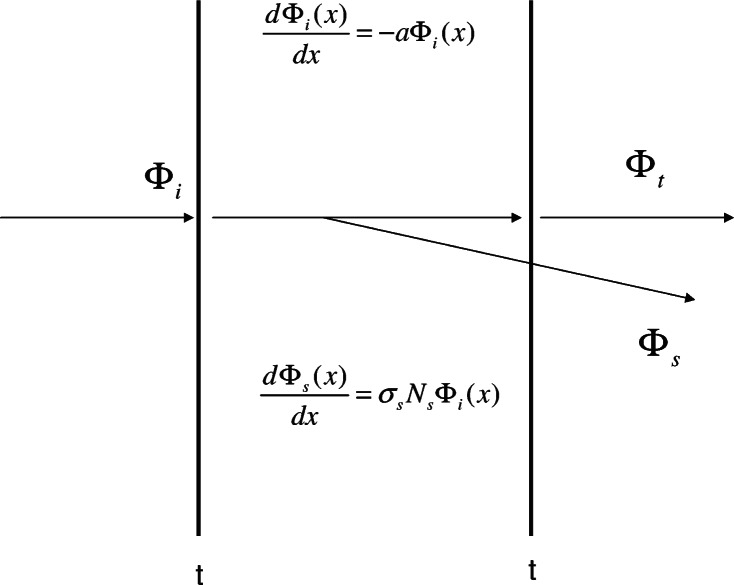
A model of the light fluxes exciting the cuvette. The incident flux is attenuated by its passage through the cuvette and exits the back of the cuvette as the transmitted flux Φ*_t_*. Along the path of the incident flux, a scatter flux arises due to scattering from particles in the analyte. The scattered flux, Φ*_s_*, exits the cuvette and some of the scattered flux may enter the IS detector. The thick vertical lines represent the cuvette walls each of which transmits a fraction *t* of the incident flux.

Consider next the response of the IS detector when a cuvette with a sample is placed in holder 1. It will be assumed that the material inside the cuvette both absorbs and scatters the light passing through it. Figure 2 shows a model of the expected response when a beam of light passes through a cuvette filled with the analyte solution (suspension). The model is reproduced in Eq. (5) and discussed in the following text

$$\begin{array}{l} \frac{d\Phi_{i}}{dx}=-a\Phi_{i}\\ \frac{d\Phi_{s}}{dx}=\sigma_{s}N_{s}\Phi_{i}. \end{array}$$ (5)

The two vertical lines in Fig. 2 represent the walls of the cuvette and the horizontal arrows represent the incident and transmitted flux. The total absorption coefficient will be written as *a* = *a_s_* + *a_m_* where *a_s_* = *σ_s_N_s_* describes the contribution of scattering to the measured absorbance, and *a_m_* = *σ_m_N_m_* is the molecular absorption coefficient. The symbols *σ_s_*, *σ_m_* represent the scattering and absorption cross section respectively. *N_s_*, *N_m_* represent the number concentrations of the scatterers and absorbers. The effect of the first cuvette wall is represented by a change in the incident flux to *t*Φ*_i_* = 10^log^*^t^*Φ*_i_* where *t* is the transmission of the cuvette wall. The first differential equation in Eq. (5) can be solved to find the flux after the beam has traversed the fluid inside the cuvette. The result is *t*Φ*_i_e^–ali^* = *t*Φ*_i_* 10^–0.434^*^al^* where *l* is the path length which will be set to 1 cm. The second cuvette wall introduces another transmission coefficient *t* giving the final transmitted flux Φ*_t_* = *t*^2^Φ*_i_* 10^–0.434^*^al^*. Next, consider the scattered flux which originates from the scattering of the incident flux by the material inside the cuvette. Solving the second differential equation in Eq. (5) gives

$$\Phi_{s}=t\frac{a_{sp}}{a_{s}+a_{m}}\left(1-10^{-0.434\left(a_{s}+a_{m}\right)}\right)t\Phi_{i}$$ (6)

where the two quantities *a_s_*, *a_sp_* are given by Eq. (7) [13]

$$\begin{array}{l} a_{s}=10^{4}N_{s}\frac{\lambda^{2}}{2\pi n^{2}}\int_{0}^{\pi }i\left(\theta \right)\sin\left(\theta \right)d\theta \\ a_{sp}=10^{4}N_{s}\frac{\lambda^{2}}{2\pi n^{2}}\int_{0}^{\Delta }i\left(\theta \right)\sin\left(\theta \right)d\theta \end{array}$$ (7)

where *n* is the index of refraction of the solution, and 10^4^ converts the area units from m^2^ to cm^2^. The quantity *a_s_* is proportional to the total scattered flux, while the quantity *a_sp_* is proportional to the scattered flux within the detector acceptance aperture which is denoted by the symbol Δ in Eq. (7). The quantity *i*(*θ*) is the differential scattering cross section averaged over the two possible polarizations. For the purpose of modeling the response, we used a software package called Far Field Mie Scattering (Valley Scientific, Inc.) to provide the values of *i*(*θ*) for specified particle and illumination conditions. (The formula in Ref. [13] defines *i*(*θ*) as the sum of the contributions from the two possible polarizations, hence the factor of 2 instead of 4 in the denominator of Eq. (7)). Note that the model summarized in Eq. (5) assumes that the attenuation is independent of the angle of scattering, and that multiple scattering is not important. Therefore the expression in Eq. (6) is an approximation. Finally the transmitted and the scattered fluxes can be written in terms of the incident flux and the properties of the cuvette to give the ratio of expected signals as shown in Eq. (8)

$$\begin{array}{l} \frac{D_{sample}}{D_{ref}}=\frac{M_{inc}\Phi_{t}+M_{inc}\Phi_{s}}{M_{ref}\Phi_{ref}}\\ \frac{D_{sample}}{D_{ref}}=t^{2}10^{-0.434\left(a_{s}+a_{m}\right)}+t^{2}\frac{a_{sp}}{a_{s}+a_{m}}\left(1-10^{-0.434\left(a_{s}+a_{m}\right)}\right)\\ \frac{D_{sample}}{D_{ref}}\equiv 10^{-A_{1}.} \end{array}$$ (8)

The value of *A*_1_ presented by the instrument can be compared directly to the function given by the model in Eq. (8). The magnification factor for the transmitted and scattered fluxes is set to the same value since both fluxes enter the IS through the same entrance aperture. The two terms in Eq. (8) represent the total flux comprised of the transmitted and scattering components. A measurement of the absorbance with buffer in the cuvette would give just the “absorbance” due to the finite transmittance at the cuvette walls, which can be used to estimate the *t*^2^ factor in Eq. (8). The relation in Eq. (8) suggests that it is not possible to obtain the scattering and molecular absorption terms simply by subtracting the buffer absorption from the measurement of total absorption. The measured absorbance should be very different for samples placed in cuvette holder 2 since the detector acceptance angle is much larger for holder 2. We discuss this case next.

### 2.2 Response of the IS Detector With Sample in Cuvette Holder 2

**Fig. 3:**
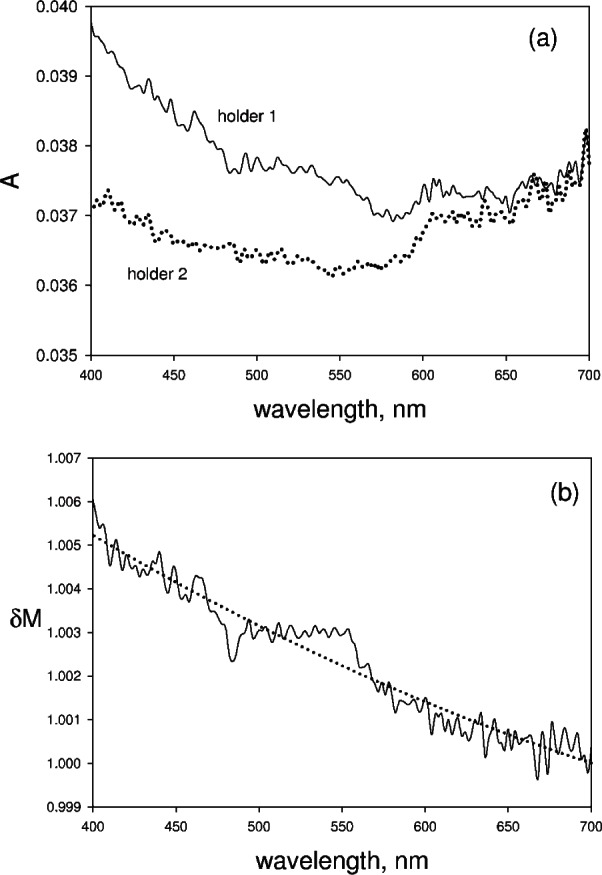
Figure 3a, the solid trace shows the measured absorbance from a cuvette filled with water and placed in holder 1. The dotted trace in Fig. 3a shows the measured absorbance when the same cuvette is placed in holder 2. The discussion following Eq. (9) in the text indicates how the two measurements can be used to estimate the change in IS magnification factor due to the presence of a cuvette at the sample entrance aperture. The solid trace in Fig. 3b shows the estimated change in magnification factor *δM* obtained from the data in Fig. 3a. The change is less than 1 %. The dotted trace in Fig. 3b is a fit to the data using a second order polynomial.

The response of the instrument to the sample in cuvette holder 2 can be modeled in the same way as the response to the cuvette in holder 1. The only changes are in the value of the instrument acceptance aperture in Eq. (7), and the magnification factor change due to the presence of a cuvette at the sample entrance port. To account for different acceptance aperture, the quantity *a_sp_* in Eq. (8) is changed to $a_{sp}^{\prime }$ where the prime indicates that the acceptance aperture is appropriate for cuvette in holder 2. When a cuvette is placed in front of the entrance aperture, the magnification factor may be different because the entrance aperture now has a finite reflectance. This fact is made explicit by writing the magnification factors with a prime in Eq. (8). The auto zero relation in Eq. (4) is not appropriate for the case of a cuvette in holder 2. It will be assumed that the magnification factors for the transmitted and scattered fluxes are equal. Differences between the two magnification factors will lead to systematic uncertainties in the final results. All of the other considerations are identical to those discussed for holder 1 so that a modified form of Eq. (8) can be used to model the response for a cuvette in holder 2

$$\begin{array}{l} \frac{D_{sample}}{D_{ref}}=\frac{M_{inc}^{\prime }\Phi_{t}+M_{inc}^{\prime }\Phi_{s}}{M_{ref}^{\prime }\Phi_{ref}}\\ \frac{D_{sample}}{D_{ref}}=\left(t^{2}10^{-0.434\left(a_{s}+a_{m}\right)}+t^{2}\frac{a_{sp}^{\prime }}{a_{s}+a_{m}}\left(1-10^{-0.434\left(a_{s}+a_{m}\right)}\right)\right)\delta M\\ \frac{D_{sample}}{D_{ref}}\equiv 10^{-A_{2}} \end{array}$$ (9)

where by definition $\delta M=\left(M_{inc}^{\prime }/M_{ref}^{\prime }\right)\left(M_{ref}/M_{inc}\right)$ where $M_{inc}^{\prime }$ is the magnification factor for the beam entering the sample aperture with a cuvette in front of it, and $M_{ref}^{\prime }$ is the magnification factor for the beam entering through the reference aperture with a cuvette placed in front of the sample aperture. The quantity $a_{sp}^{\prime }$ is larger than *a_sp_* so that the response predicted by Eq. (9) is expected to be different from the response predicted by Eq. (8). The magnitude of the factor *δM* can be estimated by measuring water filled cuvette in holders 1 and 2 after performing an auto zero function with all holders empty. To a very good approximation the absorbance and scattering of water is close to zero. Therefore comparing Eq. (8) and Eq. (9) for the case where *a_s_* and *a_m_* vanish, we obtain the relation $\delta M=10^{-A_{2}}/10^{-A_{1}}$ where *A*_1_ and *A*_2_ are the absorbencies measured in holder 1 and holder 2 respectively. The data is shown in Fig. 3a and the resulting factor *δM* is shown in Fig. 3b. The factor *δM* differs from 1 by less than 1 % over the range of wavelengths 400 nm to 700 nm. The factor is slightly greater than 1 as would be expected for a reflecting surface placed in front of the sample port. The factor *δM* will be neglected in the subsequent discussion.

### 2.3 Summation of the Measurement Model

The two equations, Eq. (8), Eq. (9), provide an explicit relation between the measured absorbencies and the fluxes that exist in the IS detector. The discussion of the model response was general and contains parameters characterizing the scattering and molecular absorption. The measurement model provides a method for extracting the apparent absorption, *a_s_*, due to scattering and the molecular absorption, *a_m_*. In the case of holder 1, the sphere accepts a small portion of the scattered flux exiting the cuvette while in the case of holder 2 the sphere accepts almost all of the flux exiting the cuvette. Fluorescence was neglected. Equation (8) can be used to describe buffer absorbance which is assumed to have no scattering (*a_s_* = 0) and no molecular absorption (*a_m_* = 0). Equation (8) suggests that for the case of a buffer, the transmission coefficient can be evaluated using Eq. (10) and the assumption of normal incidence

$$t^{2}=10^{2\log t}=10^{-A_{1}^{buf}.}$$ (10)

However, the form of Eq. (8) and Eq. (9) suggests that in general the buffer absorbance can not be simply subtracted from the sample absorbance to yield the true absorbance. The objective of the following discussion is to suggest a method for using measurements of absorbance in holders 1 and 2 to extract separate values for the apparent scattering absorption, *a_s_*, and molecular absorption *a_m_*.

## 3. Measurement of Absorbance in Scattering Suspensions

In order to test the utility of Eq. (8) and Eq. (9), measurements were performed on microspheres suspended in an aqueous solution of holmium oxide, and microspheres suspended in an aqueous solution of fluorescein. Measurements were also performed on a suspension of microalgae. Some of the suspensions were sufficiently dilute so that approximate forms of Eqs. (8) and (9) could be utilized. In the following, the approximate forms are presented and the data analyzed.

As discussed previously, to a good approximation the magnification factor is not changed appreciably when a cuvette is placed in front of the IS entrance aperture. In that case, Eq. (8) and Eq. (9) have identical form with different values of *a_sp_*. We use the approximation 10*^x^* = 1 – *x* ln (10), which is valid for *x* « 1, and obtain the following approximate relations for Eq. (8) and Eq. (9)

$$\begin{array}{l} 10^{-A_{1}}\approx 10^{-0.434\left(a_{s}+a_{m}-a_{sp}\right)}+2\log\left(t\right)\\ 10^{-A_{2}}\approx 10^{-0.434\left(a_{s}+a_{m}-a_{sp}^{\prime }\right)}+2\log\left(t\right). \end{array}$$ (11)

Equation (10) can be used to relate *t* to the measured absorbance of the buffer in the cuvette placed in holder 1. Subtracting the measured buffer absorbance from the absorbance in holder 1 and holder 2 gives the final relation between measured absorbencies and the suspension properties

$$\begin{array}{l} A_{1}-A_{{}_{1}}^{buf}\approx 0.434\left(a_{m}+a_{s}-a_{sp}\right)\\ \;=0.434\left(a_{m}+a_{s}\right)-0.434a_{sp}\\ A_{2}-A_{2}^{buf}\approx 0.434\left(a_{m}+a_{s}-a_{sp}^{\prime }\right)\\ \;=0.434a_{m}+0.434\left(a_{s}-a_{sp}^{\prime }\right). \end{array}$$ (12)

The last terms on the right side of Eq. (12), are small since to a good approximation *a_sp_* ≈ 0 for holder 1 and $a_{sp}^{\prime }\approx a_{s}$ for holder 2. Equation (12) can be used to analyze the results of measurements on dilute suspensions. In the case where the values of *a_s_* and *a_m_* are not small, a slightly more complicated analysis is required. Assuming again that in holder 1, the value of *a_sp_* is approximately 0, Eq. (8) reduces to a relatively simple relationship between the measured absorbencies and the scattering and molecular absorption

$$A_{1}-A_{{}_{1}}^{buf}=0.434\left(a_{s}+a_{m}\right).$$ (13)

Making the assumption that in holder 2, the value of $a_{sp}^{\prime }$ is approximately equal to *a_s_*, we obtain another relation using Eq. (9) with *δM* set to 1.

$$10^{-\left(A_{2}-A_{2}^{buf}\right)}=\frac{a_{m}}{a_{s}+a_{m}}\left(10^{-0.434\left(a_{s}+a_{m}\right)}-1\right)+1.$$ (14)

Equations (13) and (14) provide two relations which can be used to solve for *a_s_* and *a_m_*. Note that to first order the right side of Eq. (14) does not depend on *a_s_*. Therefore a good start is to assume a reasonable value for *a_s_*and use Eq. (14) to obtain *a_m_* at each value of the wavelength. This can be done using an algorithm which finds the zero of a specified function. The resulting values of *a_m_* at different wavelengths are inserted into Eq. (13) to obtain an estimate of *a_s_*. If needed, the new values of *a_s_* can be put back into Eq. (14) to obtain a new estimate of *a_m_*. This iterative process yields a self consistent set of values of *a_m_* and *a_s_*. The procedure works well because Eq. (14) mainly depends on *a_m_* and is not sensitive to *a_s_*. The above procedure is applicable to larger values of *a_m_* and *a_s_* and extends the analysis used in Eq. (12).

### 3.1 Mixture of a Molecular Absorber and a Scatterer

**Fig. 4:**
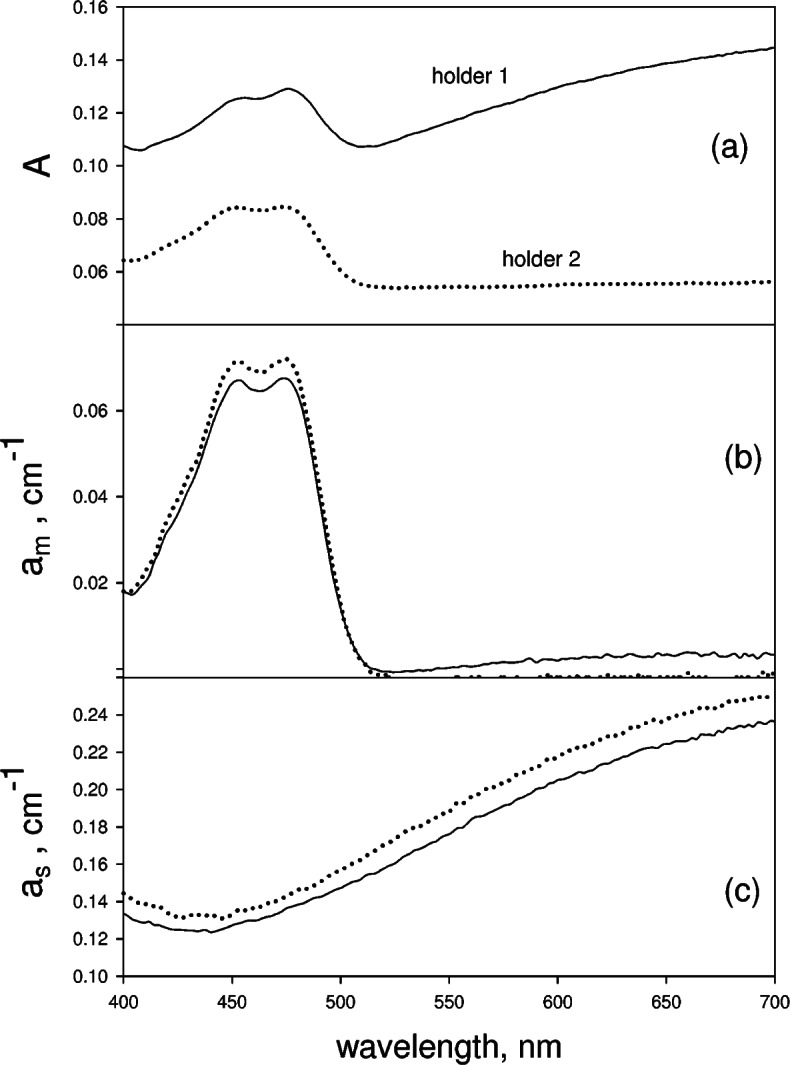
The solid trace in Fig. 4a shows the measured absorbance in holder 1 from a cuvette containing a mixture of microspheres and fluorescein in an acetate buffer. The dotted trace in Fig. 4a shows the measured absorbance when the cuvette is transferred to holder 2. The solid trace in Fig. 4b shows the derived molecular absorption coefficient using Eq. (8) and Eq. (9) and the data in Fig. 4a. The dotted trace in Fig. 4b shows the measured molecular absorption coefficient of an equivalent solution of fluorescein without the microspheres. The solid trace in Fig. 4c shows the apparent scattering absorption coefficient obtained using Eq. (8), Eq. (9) and the data in Fig. 4a. The dotted trace in Fig. 4c shows the measured apparent scattering absorption coefficient of an equivalent suspension of microspheres without fluorescein.

Figure 4a shows the measured absorbance of carboxyl modified polystyrene spheres (Bangs Laboratories, PC05N) of diameter 2.04 μm suspended in fluorescein solution. The microspheres were diluted by 10^4^ and suspended in a solution containing a 50 fold dilution of SRM 1932 (fluorescein solution) in acetate buffer. The pH of the acetate buffer is close to 5 thus minimizing fluorescein fluorescence. The solid trace in Fig. 4a shows the measured absorbance in holder 1 and the dotted trace shows the measured absorbance in holder 2. The value of absorbance (after correction for buffer) in the dotted trace at 700 nm was taken as the estimate of the contribution from scattering in holder 2. In accordance with Eq. (12), the scattering contribution, $\left(a_{s}-a_{sp}^{\prime }\right)$, was subtracted from the dotted trace to yield an estimate of *a_m_*. The estimated value of *a_m_* is shown by the solid trace in Fig. 4b. The dotted trace in Fig. 4b shows the measured absorption in holder 1 for a fluorescein solution in acetate buffer (without microspheres) where the fluorescein concentration is approximately equal to the fluorescein concentration in the suspension containing the microspheres. The correspondence between the estimated *a_m_* obtained from the suspension and the *a_m_* obtained from the equivalent fluorescein solution is good. The solid trace in Fig. 4c shows the result of subtracting *a_m_* from the solid trace in Fig. 4a (see Eq. (12)). The dotted trace in Fig. 4c is a measurement of absorption due to scattering of an equivalent suspension of microspheres in water. The values of the two traces in Fig. 4c differ slightly, however the functional form of the two traces is the same showing that only scattering is contributing. The uncertainties in the measurement of absorbance due to instrument noise were of the order of 0.001 absorbance units. There was an additional uncertainty of about 0.001 in suspension measurements due to the fluctuation of microsphere concentration. Changes in measured values due to instrument drift were negligible over a period of 10 min to 15 min, the time required to accumulate the set of data shown in Fig. 4a. However instrument drift could introduce differences of the order of 0.002 between data accumulated at times separated by hours. The systematic uncertainties due to instrument drift are difficult to characterize. The differences between the measurements displayed in Fig. 4b and Fig. 4c are of the order of 0.01, and thus are greater than the differences expected from instrumental noise and drifts. Furthermore the differences between the traces in Fig. 4b and Fig. 4c show a clear trend suggesting a small systematic bias in the analysis of the data. Nevertheless, the overall consistency of the results obtained for microspheres suspended in fluorescein solution indicate that the treatment of the data using Eq. (8) and Eq. (9) does permit the separation of contributions from scattering and molecular absorption, albeit some refinements may be necessary.

**Fig. 5:**
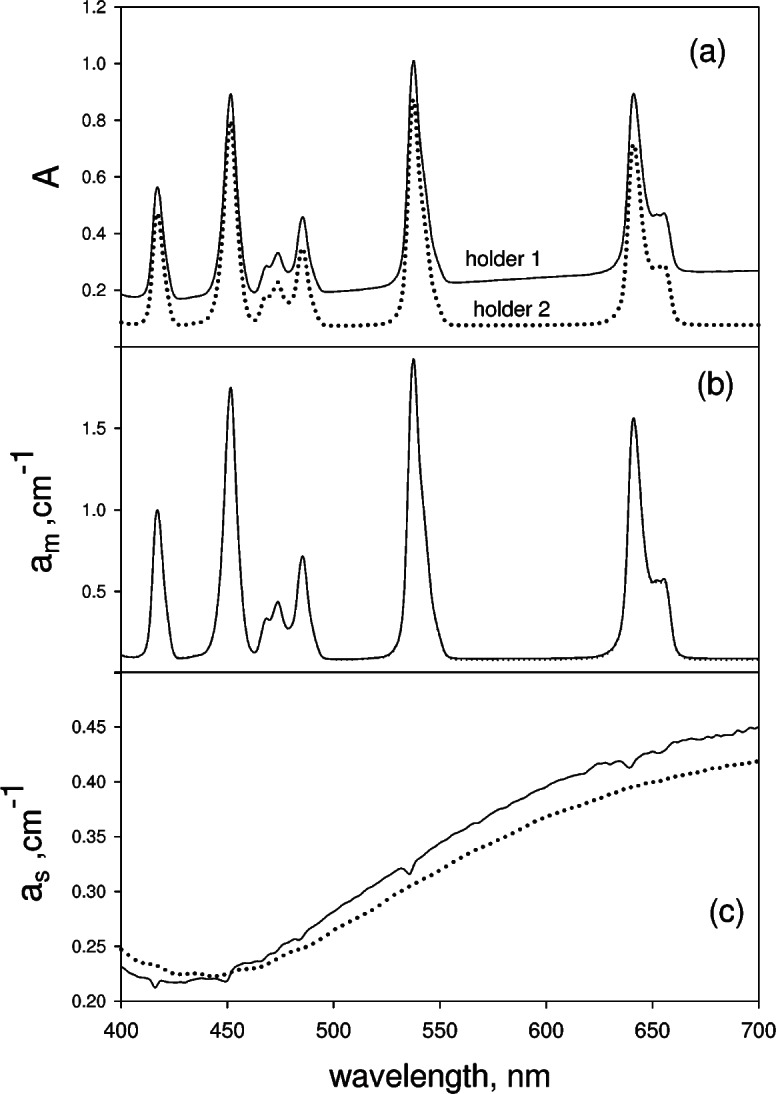
The solid trace in Fig. 5a shows the measured absorbance in holder 1 for a cuvette containing a mixture of microspheres and holmium oxide in an aqueous buffer. The dotted trace in Fig. 5a shows the measured absorbance when the cuvette is transferred to holder 2. The solid trace in Fig. 5b shows the molecular absorption coefficient obtained using Eq. (8) and Eq. (9) and the data in Fig. 5a. The dotted trace in Fig. 5b (overlaps the solid trace) shows the measured molecular absorption coefficient of an equivalent solution of holmium oxide without the microspheres. The solid trace in Fig. 5c shows the apparent scattering absorption coefficient obtained using Eq. (8) and Eq. (9) and the data in Fig. 5a. The dotted trace in Fig. 5c shows the measured apparent scattering absorption coefficient of an equivalent suspension of microspheres without holmium oxide.

Figure 5a show the results for a suspension of carboxyl modified polystyrene spheres (Bangs Laboratories, PC05N) of diameter 2.04 μm in a aqueous solution containing holmium oxide (4 % mass fraction) and perchloric acid (10 % volume fraction). The aqueous solution is equivalent to SRM 2034. The solid trace and the dotted trace in Fig. 5a show the measured absorbencies in holders 1 and 2 respectively. The absorbance at 700 nm in the solid trace in Fig. 5a was used to estimate the contribution from scattering *a_s_* in Eq. (14). Following the procedure described above yields an estimate of the molecular absorption *a_m_* shown by the solid trace in Fig. 5b. The dotted trace in Fig. 5b, which overlaps the solid trace, shows the measured absorption of an equivalent holmium solution without microsphere. The correspondence between the two traces in Fig. 5b is excellent. Following the procedure outlined in Eq. (13) and Eq. (14), the estimated *a_m_* was subtracted from $A_{1}-A_{{}_{1}}^{buf}$ to give the apparent absorption due to scattering which is shown by the solid trace in Fig. 5c. The dotted trace in Fig. 5c shows the apparent absorption measured for an equivalent suspension of microspheres in water. The overall correspondence is good suggesting that the analysis using the method outlined for larger values of *a_s_* and *a_m_* is valid. However, as discussed in conjunction with Fig. 4, the systematic differences between the two traces in Fig. 5c are outside instrumental uncertainties and indicate a small systematic bias in the analysis.

### 3.2 Properties of Microalgae Suspension

*Chlorella fusca* var. *fusca* (UTEX number 343) was obtained from the Culture Collection of Algae, University of Texas at Austin. This organism was selected according to its ecological relevance and ease of culture in the laboratory [14]. Cultures were maintained in Proteose Medium, which is the medium from Bristol’s recipe modified by H. C. Bold (2.94 mmol/L NaNO_3_, 0.17 mmol/L CaCl_2_, 0.3 mmol/L MgSO_4_, 0.43 mmol/L K_2_HPO_4_, 1.29 mmol/L KH_2_PO_4_ and 0.43 mmol/L NaCl, pH 6.8) and supplemented with 1g/L of Proteose Peptone. All cultures were started from stock cultures initiated from single alga grown on semi-solid plates of the appropriate media containing 3 % agar. Newly transferred cultures were incubated at 20 °C to 25 °C under a cool-white fluorescent lamp for 12 h and then in the dark for 12 h, allowing cells to grow until a desired population density was achieved, usually at 1,000,000 cells per mL. The possible contamination and cell density were monitored and counted with a microscope.

**Fig. 6:**
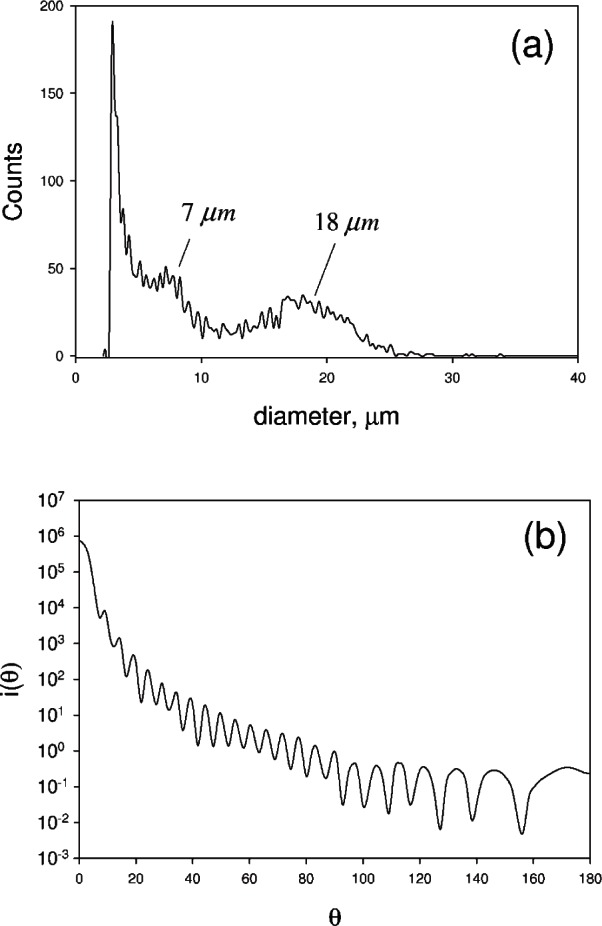
(a) The distribution of particle sizes when a diluted suspension of microalgae is passed through a Coulter Multisizer 3 particle counter. The peak at 7 μm corresponds to single microalgae passing through the orifice. The peak at 18 μm corresponds to aggregates of microalgae containing from 10 to 20 individual microalga. There may be even larger aggregates in the microalgae suspension; however the dilution and stirring in the Multisizer 3 instrument may destroy these larger aggregates. (b) Differential scattering cross section calculated for a particle of diameter 7 μm, relative index of refraction equal to 1.03, and 600 nm illumination. The scattering is mostly in the forward direction.

**Fig. 7:**
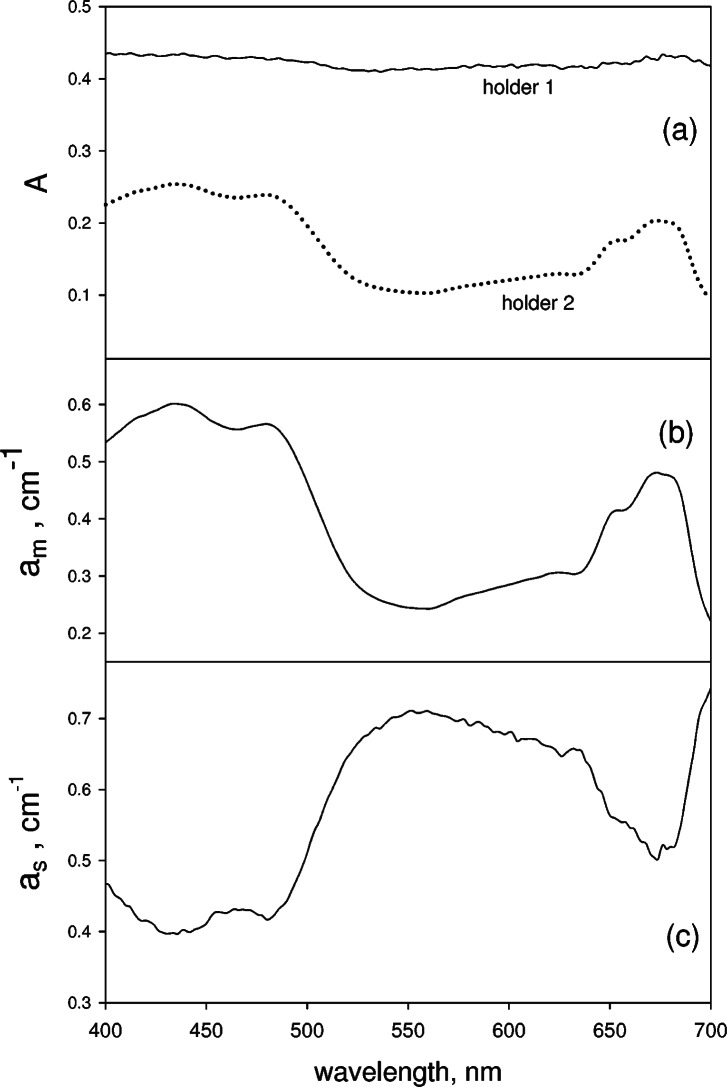
The solid trace in Fig. 7a shows the measured absorbance in holder 1 for a cuvette containing a suspension of microalgae in a growth medium. The dotted trace in Fig. 7a shows the measured absorbance when the cuvette is transferred to holder 2. The solid trace in Fig. 7b shows the molecular absorption coefficient obtained using Eq. (8), Eq. (9) and the data in Fig. 7a. The solid trace in Fig. 7c shows the apparent scattering absorption coefficient obtained using Eq. (8), Eq. (9) and the data in Fig. 7a.

Figure 6a shows the particle size distribution obtained from a suspension of microalgae passed through a Coulter Multisizer 3 particle counter. A fraction of the algae are single cells (peak at 7 μm), however a major fraction are found in aggregates of 10 to 20 cells (peak at 18 μm). The cell density determined using the Multisizer was consistent with the cell density determined by counting under a microscope. Figure 6b shows the calculated differential scattering cross section as a function of scattering angle. Mie theory appears to give a valid description of the scattering process from microalgae [15]. The calculation was performed for a wavelength of 600 nm, a diameter of 7 μm, and a relative index of refraction of 1.03 [16]. The result shows that most of the scattering occurs in the forward direction with *θ* ≤ 20. Inserting *i* (*θ*) displayed in Fig. 6b into Eq. (7), and using a cell concentration of 10^6^ cm^–3^, gives *a_s_* = 0.41. This value is smaller than the value *a_s_* = 0.65 derived from measurements (see Fig. 7c). The difference is not surprising because the suspension contains a large number of aggregated microalgae cells which are expected to scatter differently. Furthermore the relative index of refraction could be different for this specific species of microalgae.

### 3.3 Measurement of Absorbance of Microalgae Suspension

During the measurements of absorbance of a suspension of microalgae in cuvette placed in holders 1 and 2, the suspension was stirred to keep the suspension from settling. Measurements carried out after the lapse of about half hour indicate that the absorbance values are very close indicating that settling has not occurred. Fluorescence from chlorophyll molecules is not a major source of uncertainty since the quantum yield from algae is found to be of the order of 0.02 [17]. Furthermore the fluorescence emission is isotropic resulting in negligibly small fluorescence flux into the IS detector. The solid trace in Fig. 7a shows the absorbance of microalgae suspension measured in holder 1 while the dotted trace shows the absorbance measured in holder 2. Clearly there is a large difference in the measured absorbencies in the two holders. The procedure outlined in Eqs. (13) and (14) was used to analyze the data in Fig. 7a. In all cases, the measured absorbance from the growth medium (buffer) was subtracted. The solid trace in Fig. 7b shows the result for *a_m_* and the solid trace in Fig. 7c shows the resulting *a_s_*. There is substantial structure in the result for *a_s_* and the structure is a mirror image of the absorption spectrum shown in Fig. 7b. Although it is not possible to compare the values of *a_s_* and *a_m_* to independent measurements of scattering and absorption, it can be expected that the traces shown in Fig. 7b and Fig. 7c are representative of the “true” values with some qualifications as discussed in connection with Fig. 4 and Fig. 5.

Measurements were performed in diluted suspension of microalgae so that all measured absorbencies were less than 0.2 and the measurements could be analyzed using Eq. (12). The results were similar to those shown in Fig. 7. Therefore neither the degree of approximation in the analysis nor the microalgae concentration seems to influence the final results. Measurements were carried out on microalgae suspensions with reduced illumination. The illumination level was reduced by inserting neutral density filters in the incident beam. The results for 1 % illumination were the same as those for 100 % illumination.

## 4. Discussion

The results for mixtures of microspheres and absorbing solutions, shown in Fig. 4 and Fig. 5, suggest that the analysis based on Eq. (12) or Eqs. (13) and (14) gives a reasonably good separation of the contributions from molecular absorption and scattering. The two systems studied consisted of a mixture of nonabsorbing microspheres and absorbing chromophore molecules in solution. Therefore it is safe to assume that the scattering process, which arises from the microspheres, and molecular absorption process, which arises from the chromophores, were completely independent. In the case of microalgae, the absorbing chromophores are packed inside the cell. In this case, the contributions from scattering and molecular absorption are not independent. In fact, it is expected that microalgae scattering and molecular absorption are highly correlated. The description of scattering given by Eq. (6) and Eq. (7) is applicable to microspheres which contain an absorbing material (e.g., algae cells with chlorophyll). Using this scattering formalism it is possible to describe the total energy loss from a beam of light passing through a cell suspension as arising from two processes: the elastic scattering by the cell interface and molecular absorption by the chlorophyll molecules inside the cell [18]. The crux of the matter is that the measurement model in Eq. (5) is equally applicable to microalgae and to mixtures of independent scatterers and absorbers. Therefore the microalgae response shown in Fig. 7b, and Fig. 7c must be of equal validity as the response found for mixtures of microspheres and chromophores.

The presence of strong spectral features in the measured scattered light from microalgae suspensions has been noted previously [19]. Early work in this area was reviewed by Butler [20]. The strong spectral features in the scattered light (see Fig. 7c) have been called “selective scattering” and it was noted the selective scattering effect depended on the acceptance aperture of the instrument used in the measurement. Recent attempts to compensate for nonselective and selective scattering and to obtain the correct absorption spectrum are described by Merzlyak [21]. The selective scattering response originates from the large enhancement of various molecular transition matrix elements whenever the energy of the incident photons matches the energy of a molecular electronic transition. Therefore the expression “selective scattering” is synonymous with “resonance light scattering.” Resonance light scattering has been widely observed in Raman scattering. However the resonance effects are equally applicable to luminescence and Rayleigh scattering [22]. Resonance scattering should be minimal in the case of mixtures of microspheres and chromophores since scattering from the individual chromophores is negligible compared to scattering from the microspheres. In the case of microalgae, scattering and absorption are highly correlated thus leading to the observation of resonance scattering. Naqvi made the assumption that scattering and molecular absorption are two parts of a function characterizing the response of cells to light [19]. Applying the Kramers-Kronig relation to the absorption spectrum, Naqvi obtained the scattering spectrum very similar to the pair shown in Fig. 7b, and Fig. 7c. The chlorophyll molecules in algae are packed tightly and may form arrays of interacting chromophores. Such interacting arrays may exhibit additional enhanced scattering [23]. The connection between resonance scattering and chromophore packing inside the cell was used by Bialek to probe the structure of chloroplasts [24]. We end with a few qualifying comments regarding the interpretation of the data in Fig. 7c. If the resonance scattering contribution has the same angular distribution as the nonresonance Mie scattering then the resonance scattering should enhance the values of *a_s_* and not decrease them as shown in Fig. 7c. However if the resonance scattering is highly peaked in the forward direction, then the instrument will not be able to distinguish the resonance scattering from transmitted light and interpret the contribution from resonance scattering as a reduction in absorbance. In this case the measured values of *a_s_* will decrease as in Fig. 7c, and the resonance scattering contribution can be estimated from the difference of the values of *a_s_* at resonance and nonresonance wavelengths. A possibility exists that a third phenomenon such as stimulated emission is occurring and reducing the observed absorbance. However, stimulated emission is expected only at large illumination power. It is likely that an additional measurement is needed to interpret the data in Fig. 7c in terms of a molecular process.

## 5. Conclusion

An analysis of the measurement process in a spectrometer with an integrating sphere (IS) detector lead to a procedure for separating the contributions to the measured absorbance due to scattering and the molecular absorption. The analysis hinges on the interpretation of absorbance measured for a cuvette placed in two holders in the spectrometer. Holder 1 is the normal position, and holder 2 places the cuvette at the entrance aperture of the IS detector. Eq. (8) and Eq. (9) give the relationship between the measured absorbencies (*A*_1_, *A*_2_) and the analyte properties (*a_s_*, *a_m_*). Approximate forms of the two equations are used to analyze several systems consisting of scatterers and absorbers. The results suggest that it is possible to separate the absorption and scattering contributions. Furthermore, the results with microalgae suggest that the two quantities *a_s_*, *a_m_* are independent characteristics of the microalgae suspension. The quantity *a_m_* gives information about the electronic states of the absorbing chlorophyll molecules, while the quantity *a_s_* may provide information about the packing of the chromophores inside the microalgae cells. Further work is needed to clarify the systematic uncertainties inherent in the measurement model. The most significant of these uncertainties are the estimates of the partial cross sections *a_sp_* and $a_{sp}^{\prime }$ in Eq. (8) and Eq. (9). Both estimates depend on the instrument configuration as well as the angular distribution of the scattered radiation.

## Footnotes

1 Certain commercial equipment, instruments, or materials are identified in this paper to foster understanding. Such identification does not imply recommendation or endorsement by the National Institute of Standards and Technology, nor does it imply that the materials or equipment identified are necessarily the best available for the purpose.
